# Physiologic Insulin Resensitization as a Treatment Modality for Insulin Resistance Pathophysiology

**DOI:** 10.3390/ijms23031884

**Published:** 2022-02-08

**Authors:** Frank Greenway, Brian Loveridge, Richard M. Grimes, Tori R. Tucker, Michael Alexander, Scott A. Hepford, Justin Fontenot, Candi Nobles-James, Carol Wilson, Adam M. Starr, Mohammed Abdelsaid, Stanley T. Lewis, Jonathan R. T. Lakey

**Affiliations:** 1 Clinical Trials Unit, Pennington Biomedical Research Center, Louisiana State University, Baton Rouge, LA 70808, USA; frank.greenway@pbrc.edu; 2 Diabetes Relief, Layton, UT 84041, USA; brian@diabetesrelief.com; 3 Department of Internal Medicine, McGovern Medical School, University of Texas Health Science Center, Houston, TX 77030, USA; richard.m.grimes@uth.tmc.edu; 4 Department of Development and Cell Biology, University of California Irvine, Irvine, CA 92617, USA; trtucker@uci.edu; 5 Department of Surgery, University of California Irvine, Orange, CA 92868, USA; michaela@hs.uci.edu; 6 Well Cell Global LLC, Houston, TX 77079, USA; scott@wellcellglobal.com; 7 Lafayette Arthritis and Endocrine Clinic, Lafayette, LA 70506, USA; justinfontenotmd@gmail.com; 8 Division of Endocrinology, Mercer University School of Medicine, Macon, GA 31207, USA; nobles-james_cn@mercer.edu; 9 Well Cell Support LLC, Houston, TX 77079, USA; carol@wellcellsupport.com; 10 Department of Orthopedic Surgery, Asheville Orthopedic Associates P.A, Asheville, NC 28801, USA; adam@kalamountain.com; 11 Biomedical Sciences Department, Mercer University School of Medicine, Savannah, GA 31404, USA; abdelsaid_ma@mercer.edu; 12 Eselle Health, Inc., La Jolla, CA 92037, USA; slewis@esellehealth.com

**Keywords:** insulin resistance, diabetes, metabolic disorder, obesity, insulin infusion, physiologic insulin resensitization, PIR, treatment modality, neuropathy, nephropathy, retinopathy, cardiovascular disease, chronic kidney disease, CKD

## Abstract

Prevalence of type 2 diabetes increased from 2.5% of the US population in 1990 to 10.5% in 2018. This creates a major public health problem, due to increases in long-term complications of diabetes, including neuropathy, retinopathy, nephropathy, skin ulcers, amputations, and atherosclerotic cardiovascular disease. In this review, we evaluated the scientific basis that supports the use of physiologic insulin resensitization. Insulin resistance is the primary cause of type 2 diabetes. Insulin resistance leads to increasing insulin secretion, leading to beta-cell exhaustion or burnout. This triggers a cascade leading to islet cell destruction and the long-term complications of type 2 diabetes. Concurrent with insulin resistance, the regular bursts of insulin from the pancreas become irregular. This has been treated by the precise administration of insulin more physiologically. There is consistent evidence that this treatment modality can reverse the diabetes-associated complications of neuropathy, diabetic ulcers, nephropathy, and retinopathy, and that it lowers HbA1c. In conclusion, physiologic insulin resensitization has a persuasive scientific basis, significant treatment potential, and likely cost benefits.

## 1. Introduction

The prevalence of type 2 diabetes has increased from 2.5% of the US population in 1990 and constituted 10.5% of the total US population or 13.0% of US adults in 2018 [1]. This is an astounding 320% increase in 28 years. In addition, a third of American adults, approximately 88 million people, have prediabetes [2]. Patients with pre-diabetes will progress to diabetes at a rate of 5–10% per year [3]. While, currently, we have a major diabetes-associated health care problem, a surge of new diabetes patients looms. An already overburdened health care system can expect major increases in the long-term complications of type 2 diabetes, which include neuropathy, retinopathy, nephropathy, ulcers, amputations, and atherosclerotic cardiovascular disease. Diabetes is a very expensive disease that cost the US economy in 2017 an estimated $327 Billion in direct medical costs and another $90 Billion in lost productivity. Given that the annual direct medical cost for treating diabetes increased from $245 Billion to $327 Billion per year between 2012 and 2017 [4], it is not unreasonable to assume that the cost of treating diabetes in 2021 will approach $400 Billion. This expenditure is high and increasing because it is directed toward a progressive disease for which therapy is designed to slow the progression of diabetes-associated conditions until death occurs. 

We first turned our attention to the most current guidelines from the American Diabetes Association (ADA), which has recommend the use of medications classified as sodium-glucose cotransporter 2 inhibitors (SGLT2is) and glucagon-like peptide 1 receptor agonists (GLP-1 RAs) for individuals with T2D on Metformin AND diagnosed with EITHER heart failure (HF) OR chronic kidney disease (CKD) [5], as clearly discussed by Colling, et al. [6]. They conclude that “the introduction of SGLT2is and GLP-1RAs has led to rapid changes in recommendations for the medical management of T2D” [6]. Moreover, Colling, et al. detail classes of patients for whom preferential use of GLP-1RAs and SGLT2is is and is notrecommended, and we urge a thorough review by practitioners [6]. The ADA’s “Standards of Medical Care in Diabetes” was originally approved in 1988 and the work is updated annually, most recently in December of 2020. Considerations and requirements are discussed in “Introduction: Standards of Medical Care in Diabetes—2021” [7]. 

We also note that the Food & Drug Administration (FDA) has published a March 2020 Update to its Drug Safety Communications from 2015 [8]. Further, The American College of Cardiology published on January 19, 2021, an expert analysis by Rishav Adhikari and Michael Blaha, who found that (1) uptake of these cardioprotective drugs in 2020 remained low; (2) cardiologists account for a minute percentage of prescribing for these drugs, even though their primary benefit is cardiovascular risk reduction; and (3) barriers to adoption by cardiologists include lack of knowledge about these medications and cardiologists’ perception that diabetes care is not their responsibility [9]. 

Clearly, costs will rise, side effects and FDA warnings will always be prevalent, and disability will continue to occur. However, there is a complimentary treatment that reverses the major underlying pathophysiologic mechanism that leads to T2DM with far fewer negatives, if any. This treatment is effective because it directly reverses insulin resistance (IR), not just the individual manifestations of diabetes. Moreover, unlike other medications cited, side effects are minimal, if at all. For nearly forty years, researchers and clinicians have utilized a treatment modality that dynamically administers periodic infusions of insulin that bio-mimic the non-diabetic’s natural secretions and rest periods of insulin from the pancreas [4]. There have been multiple articles describing this treatment approach that have reported efficacious clinical outcomes on various diabetic comorbidities. Although effective, the exact cellular and molecular mechanisms behind the use of periodic infusions of insulin was undetermined. This article has two purposes: (1) To explore insulin resistance pathophysiology and (2) to review literature for clinical outcomes and molecular mechanisms that support the use of physiologic insulin resensitization as an effective treatment modality to address insulin resistance, of which diabetes and its complications are the most common results.

## 2. The Rise of Type 2 Diabetes

In general, two main hypotheses were generated for the etiology and pathogenesis of type-2 DM. The first hypothesis is that the development of insulin resistance (IR) is behind type 2 diabetes. IR causes hyperglycemia, which is the cause of most diabetic complications. IR also causes hyperinsulinemia, i.e., the over-production of insulin by βbeta cells. Over time, beta cells will be exhausted and die, which leads to further disease progression and more hyperglycemia. The second hypothesis concerns the defect in insulin secretion, which is well reported in type 2 diabetes.

As stated, type 2 diabetes is driven by IR. A common perception of type 2 diabetes progression is that the IR is countered by increasing insulin secretion until the beta cells in the pancreas can no longer keep up with the insulin demand and die progressively of exhaustion [3]. Another possible sequence of events is that the IR could begin with a defect in insulin secretion. A defect in the first phase of insulin secretion is well known to be an early characteristic of type 2 diabetes [10,11]

Therefore, an alternative explanation for the progression of type 2 diabetes is that impaired insulin secretion is a corollary to the progression of obesity, thus, triggering a cascade leading to islet cell destruction and the long-term complications of type 2 diabetes. In this manuscript, we offer the hypothesis that it is an insulin secretion defect that drives the IR responsible for the pathophysiology of type 2 diabetes.

## 3. Physiologic Hormone Secretion 

Hormones that are released in an oscillatory pattern have receptors that are physiologically designed to bind the ligand, bring it into the cell, separate the receptor from its ligand and return the receptor to the cell surface. This rest period, or trough, of these oscillations gives sufficient time for this physiologic sequence to take place prior to the next peak in this cycle. When hormone receptors designed to respond to pulses of its ligand are exposed to constant stimulation with the hormone, the receptors down-regulate and become “tolerant” or “resistant” to stimulation. The amount of down regulation can be variable; for example, the gonadotropin-releasing hormone receptor is an example of a receptor that is particularly sensitive to down-regulation from a constant stimulation by its ligand. Leuprolide is a long-acting agonist of the gonadotropin-releasing hormone receptor that is given as an injection and stimulates the receptor continuously for 1–3 months. This receptor agonist down-regulates the receptor to the extent that the sex hormone secretion is blocked. In fact, leuprolide is used to block puberty or to achieve a chemical castration in the treatment of hormone-sensitive cancer [12].

### 3.1. Physiologic Insulin Secretion

**Figure 1:**
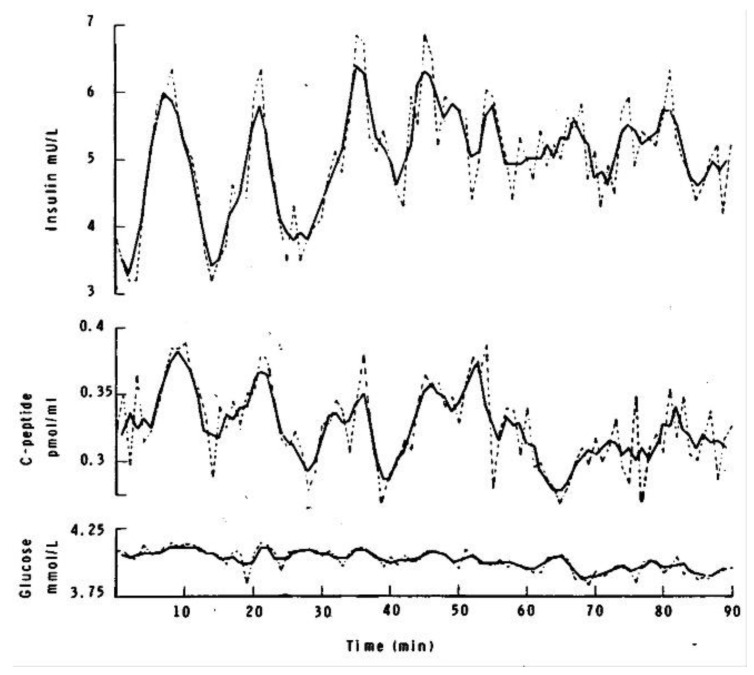
A three-minute moving average (continuous line) of the fasting plasma insulin, C-peptide and glucose concentrations taken at one-minute intervals. The dashed line shows the “unsmoothed” data. Smoothing reduces the rapid fluctuations, which are probably due to “noise,” and also blunts the amplitude. The simultaneous insulin and C-peptide cycles disappear after 50 min. Reproduced from [13].

Physiologic insulin consists of discrete oscillatory secretions and distinct rest periods to stimulate ligand/receptor activation. The beta cell secretes insulin with a dynamic periodicity of 4–8 min, and most commonly 5–6 min, based on the body’s demands (Figure 1) [13]. 

Beta cells in the islets are in close contact to one another, which allows islets to secrete insulin in such a pattern, but it requires a network of autonomic nerves to allow the pancreatic beta cells to coordinate this dynamic profile [14]. The insulin pulses are responsible for timing of dynamic glucagon secretion that occur normally anti-synchronous from the cycling of insulin [15]. Measuring the cycling of insulin is made more difficult by the fact that the beta cells secrete the insulin into the portal circulation and the concentrations in the portal vein are five times higher than in the peripheral circulation due to hepatic extraction [16]. The insulin receptor in its upregulated state senses a basal level of insulin that is about 30% of the total, and 70% is due to pulses secreted over that baseline. The increase in insulin at meals is accounted for by an increase in the amplitude of the insulin spikes and not a change in the time between oscillations. Although there is a high rate of clearance of insulin by the liver, C-peptide is secreted in an equimolar ratio to insulin, but C-peptide reaches the peripheral circulation without hepatic clearance. The deconvolution method of measuring the spike frequency from the peripheral blood depends on the differential kinetics of insulin and C-peptide [16]. Insulin resistance (IR) is associated with a reduction in the amplitude of insulin cycling and an increase in the basal level of insulin, creating more of a constant rather than oscillatory pattern of insulin stimulation of its receptor. Although obesity is associated with an increase in beta cell mass, by the time type 2 diabetes develops, there has been a 65% reduction in beta cell mass due to apoptosis associated with increased levels of amylin that is secreted in equimolar amounts to insulin [17]. This reduced beta cell mass loses the ability to maintain insulin oscillation, and a reduction in glucose-stimulated insulin secretion leads to hyperglycemia. The peripheral level of insulin is higher with an increased ratio of proinsulin to insulin, but the peaks in insulin secretion are not as high as in people with normal glucose tolerance. The lack of insulin pulses decreases hepatic clearance of insulin leading to peripheral hyperinsulinemia [18]. The insulin receptor can bind insulin in two ways, with high affinity and with low affinity. The affinity decreases as the insulin levels increase. Thus, the changing of receptor affinity for insulin is another property that contributes to hyperinsulinemia [19].

### 3.2. Insulin Sensitivity and Physiologic Secretion

The importance of oscillatory secretion signals in insulin sensitivity has been demonstrated in several ways. In patients with type 2 diabetes, an overnight infusion of somatostatin gave the beta cell a rest from constant stimulation by insulin and restored insulin pulse mass and normal insulin secretion [20]. Hepatic IR in dogs was achieved with a constant infusion of insulin that produced a 50% increase in the portal vein level of insulin [21]. Even more convincing was a study in which physiologic insulin delivery in patients with type 1 diabetes was compared to a constant infusion of insulin. A euglycemic insulin (1 mU/kg/min) clamp was performed on two occasions. On one occasion, it was infused continuously and, on the other occasion, it was infused for 3 min followed by a 7-min rest period. Despite a 40% reduction in the insulin dose, the suppression of hepatic glucose output was the same. When the total amount of insulin infused was held constant, the hepatic glucose output was 25–30% less in the cyclical condition [22]. It has also been shown in people with normal glucose metabolism that the dynamic pattern of insulin secretion enhances peripheral glucose uptake more than a continuous infusion [23].

## 4. Mechanisms of Insulin Resistance

Normally, insulin is secreted in a physiologic pattern, mediated by a pancreatic neuronal network connecting cells residing in the islets of Langerhans [24,25]. Dysfunctional insulin rhythmicity can occur from a variety of insults (i.e., obesity, auto-immune disorders, toxins, trauma, stress etc.) that lead to inflammation of this network. When physiologic patterns of insulin secretion are disrupted, beta cells secrete insulin asynchronously. As a result of constant ligand/receptor exposure, a negative feedback loop downregulates insulin receptor responsiveness. In addition, lack of physiologic peaks and troughs leads to refractory delays in receptor activity. Finally, disrupted pulsation leads to unopposed glucagon levels, which decrease transcription of insulin receptors [26].

### 4.1. Implications for Treatment

A genetic aspect to insulin resistance (IR) involves abnormal insulin signaling and leads to type 2 diabetes. The children of parents with type 2 diabetes have IR that leads to type 2 diabetes later in their adulthood. These children exhibit higher levels of insulin and beta-cell dysfunction [27]. Even people who are just close relatives of people with type 2 diabetes often have impaired insulin oscillatory patterns [28]. 

### 4.2. Diet

From a dietary perspective, the Framingham study suggests that diets with a lower glycemic index are associated with greater insulin sensitivity [29]. A low carbohydrate diet is associated with a reduction in IR compared to a high carbohydrate diet [30]. The improvement in insulin oscillation was proportional to residual insulin sensitivity after weight loss [31]. This is, and has been, an approach that targets a reduction in a precursor of the disease manifestations and not just a single symptom. However, it has proved very difficult to obtain patient cooperation in adopting and maintaining an appropriate diet that minimizes carbohydrates and results in achieving and maintaining weight loss [32,33]. 

### 4.3. Medication

From the medication perspective, as one might predict for a disease driven by IR, insulin sensitizing medication such as metformin or the thiazolidinediones prevent or restore the abnormal insulin secretion associated with IR [34,35]. Repaglinide and glucagon-like peptide-1 agonists increase the insulin peak amplitude without affecting oscillatory frequency [36]. When people with diabetes require insulin, it is given as a subcutaneous injection in a manner that exposes the insulin receptor to a constant level of insulin. Interestingly, some of the common ways of measuring insulin sensitivity are based on fasting insulin and glucose values such as homeostatic model assessment for insulin resistance (HOMA-IR) [37], which may underestimate the role that physiologic insulin cycling plays in IR. All the medications described above deal with controlling the ***effects*** of existing diabetes, and not on treating its underlying ***causes***. 

As the occurrence of diabetes continues to rise along with the ballooning costs of treatments, pharmaceutical companies continue to seek proprietary compounds for development. In recent years, the US FDA has approved several drugs with novel mechanisms of action. These include GLP-1 agonists, Dipeptidyl peptidase-4 (DPP-4) inhibitors and sodium-glucose transport protein 2 (SGLT2) inhibitors. Thiazolidinediones’ such as rosi- and pioglitazone are the only approved insulin sensitizing drugs that use insulin sensitization as the only mechanism. Physiologic insulin resensitization (PIR) is a true sensitizing strategy, as well. Rosiglitizone costs about $180–$190 a month but gives an increase of fat cells that fill with fat and increase obesity and therefore include cardiovascular safety concerns.

Although this review was written to focus on approaches that are true insulin sensitizers, such as insulin and the thiazolidinediones, glucagon-like peptide-1 (GLP-1) agonists and sodium-glucose cotransporter-2 (SGLT-2) inhibitors, through their independent mechanisms, have shown a positive effect on insulin resistance. The interested reader can refer to recent reviews on the molecular mechanisms of action of these medications [38,39], since the subject is beyond the scope of the present review. 

It is encouraging to see reductions in major cardiovascular endpoints and positive data for those suffering with renal complications of diabetes. However, the magnitude of such benefits from these novel drugs are limited in scope, while many patients are unable to tolerate due to material adverse side effects [40,41]. In addition, recent guidelines published 5 March 2018, from the American College of Physicians (ACP) on diabetic management outline that over-aggressive HbA1c control can be counterproductive and harm patients due to complications of hypoglycemia and other untoward effects. This evidence-based review includes data from the landmark ACCORD trial that was terminated prematurely, because intensive glycosylated hemoglobin management led to increased morbidity and mortality. As such, ACP guidelines now target a HbA1c between 7–8%, rather than previous targets of 6.5–7%. Thus, the standard of practice for diabetic management is in flux, highlighting the need to modify treatment modalities for optimal clinical outcomes [42]. Another important development was the reclassification by the FDA of insulin to the biologic regulatory framework in March of 2020, highlighting the physiologic importance of this hormone peptide in regulating carbohydrate metabolism [43]. 

### 4.4. Physiologic Insulin Resensitization

An alternative approach to counter IR in the euglycemic clamp of constant insulin infusion would be to reintroduce physiologic insulin delivery. This has been done by inserting an intravenous access connected to a precision infusion pump that can be programmed to dynamically deliver physiologic insulin typical of normal glucose metabolism. A more detailed description of physiologic insulin can be described as periodic cycling of up to 3 IU of regular insulin infused dynamically every 4–8 min (usually 5–6 min), based on the body’s utilization for 2 to 4 h based on an individual patient weekly basis. Oral glucose is given to simulate a meal and to keep blood glucose in a therapeutic and safe range. Patients are observed until glucose is stable after the dynamic insulin infusion is administered [44]. The mechanism of PIR is directed at the pathophysiology of IR, found in type 2 diabetes. Through upregulation of the insulin receptor/ligand complex, it may be possible to bio-modulate physiologic response in a beneficial manner. Peripheral administration of IV insulin in a rhythmic pattern would then be able to replace lost physiological signals critical to cellular glucose metabolism. With improved ability to drive glucose into the mitochondrial oxidative phosphorylation cascade, improved energy production (in the form of ATP) could then occur. As such, energy depleted tissues would have the building blocks necessary to undergo healing, repair, and cellular restoration [45].

Physiologic insulin resensitization requires a pulsatile insulin delivery comparable to that of a healthy pancreas. Understanding this type of insulin delivery is necessary; over the years, scholars have studied and reported on this concept. Notably, in 2012, researchers Matveyenko et al. reported that pulsatile insulin delivery into the systemic circulation is more efficacious than constant insulin infusion [46]. The Matveyenko study also found that the timing of the insulin receptor is perfectly suited to entrain to the episodic delivery of insulin via the sinusoids directly to hepatocytes, and they concluded that hepatic insulin signaling is delayed and impaired when insulin is delivered in a nonpulsatile manner [46]. 

Ten years earlier, Porksen, et al., discussed pulsatile insulin secretion and reported that, in type 2 diabetes mellitus, both IR and impairment of insulin secretion characterizes the metabolic problem of the disease. High frequency of insulin oscillations in these patients corresponds to serial secretory insulin bursts [47]. 

This treatment approach has been reported to achieve physiological insulin concentrations in the portal vein based on animal work [48]. This treatment, with some variation on the amount of insulin and treatment frequency, has been evaluated in case series and clinical trials that are reviewed herein. 

## 5. Studies of Physiologic Insulin Resensitization (PIR) Treatment

### 5.1. Foot Ulcer and Peripheral Neuropathy

Tucker et al. described two cases in which symptoms of diabetic neuropathy resolved with PIR. One was a 73-year-old male who displayed slow wound healing, erectile dysfunction, and numbness in his feet with a foot ulcer. Intermittent treatment with PIR achieved wound healing, numbness resolution, and a decrease in his insulin requirement from 120 to 28 units per day. The second was a 74-year-old female who experienced slow wound healing, a foot ulcer, weight gain, stage 4 chronic renal disease, numbness, pain, and tingling in her lower extremities. Over several months of receiving insulin administered in a physiologic manner, she experienced wound healing, improved sensation, and discontinuance of the gabapentin formerly taken for neuropathy pain. She also lost 15 kg, her daily insulin requirement dropped from 60 to 25 units per day, and her HbA1c dropped from 9.9 to 7.1% [49]. In another study Elliott et al. described a case series of 5 patients who were treated with physiologic insulin 1 h 3 times a day up to 5 days a week. The mean time to complete healing of foot ulcers was predicted from the literature to be 133 days, but the wounds in the 5 patients healed in a mean of 84 days [50]. The 37% reduction in the healing time was interpreted as providing a significant cost savings [51]. Dailey et al. randomly allocated 19 patients (12 men, 7 women) to either standard diabetic insulin-based care or to that care and additional day per week of 3 sessions of physiologic insulin over an 8-h period. When compared to baseline perceptions, patients receiving physiologic insulin reported significant improvement in diabetic nephropathy when compared to the control group (*p* = 0.0144) [52]. Eliott et al. reported on a study of 412 patients, 76% of whom experienced painful diabetic neuropathy and who were treated with 3 h of physiologic insulin per week for 3 months. Of those with painful diabetic neuropathy, 142 (47.5% experienced complete resolution of pain, 136 (45.5%) experienced partial resolution of pain and 21 (7%) experienced no improvement [51]. 

### 5.2. Diabetic Nephropathy

Villaverde et al. described three cases of chronic kidney disease, one in a patient with diabetes and two with pre-diabetes, that improved in response to physiologic insulin resensitization over 5–6 months. The estimated glomerular filtration rate (GFR) increased from 33, 34 and 54 cc/min to 55, 42 and 74 cc/minute, respectively. Blood urea nitrogen and creatinine improved from means of 27 and 1.7 mg/dL to 13 and 1.2 mg/dL, respectively. Not only is reversal of chronic kidney disease difficult to accomplish, but delaying renal replacement therapy is also associated with significant economic savings [53]. Manessis et al. reported an uncontrolled series of 17 patients with type 2 diabetes of greater than 2 years duration and stage 3 chronic kidney disease (GFR 30–60 cc/min) treated with weekly dynamic physiologic insulin for 3 months. The GFR increased by 12% from baseline (47.6 ± 10 cc/min to 53.3 ± 11.9, *p* < 0.01), creatinine decreased by 7% from baseline (*p* < 0.05), and systolic blood pressure decreased by 6% from baseline (*p* < 0.05) [54]. Quach and Manessis conducted a trial of 17 patients with chronic kidney failure. Patients received a total of 10 physiologic insulin infusion procedures over three months. GFR improved by an average of 10.8% and creatinine decreased by 6.8% [55]. Dailey et al. compared two randomly assigned groups of 49 patients who received either intensive diabetes treatment only (26) or intensive treatment plus physiologic insulin infusions (23). Creatinine clearance (CrCl) declined significantly in both groups, as expected, but the rate of CrCl decline in the group receiving physiologic insulin (2.21 ± 1.62 mL/min/yr) was significantly less than in the control group (7.69 ± 1.88 mL/min/yr, *p* = 0.0343) [52]. An overall summary of the physiologic insulin resensitization effect on diabetic nephropathy is provided in Table 1.

### 5.3. HbA1c 

Tucker et al. reported a 74-year-old female who presented with numerous complications after 20 years of T2D that included slow wound healing, foot ulcers, kidney disease, neuropathy, and hypertension. Her comparisons before and after PIR treatment included HbA1c reduction from 9.9 to 7.1, improved wound healing and discontinuance of Gabapentin for neuropathy, and discontinuance of her Humalog completely [49]. 

Aoki et al. treated 20 patients with brittle type 1 diabetes for periods of 7–71 months with physiologic insulin and the mean HbA1c declined from 8.5% to 7.0% [56]. Aoki reported on another study of 31 patients with type 1 diabetes, many with diabetes complications, who were controlled on a physiologic insulin regimen with injections administered 4 times a day. These patients were treated with additional oscillatory intravenous insulin for 1 h during meals 3 times a day, 1 day a week, for 7 to 71 months. The HbA1c fell from 8.5% to 7.0%. Major hypoglycemic reactions fell from 3 to 0.1 per month, and minor hypoglycemic reactions fell from 13 to 2.4 per month [44]. 

### 5.4. Cost Reduction

All the studies above reflected reduced burden of disease. However, they did not study the actual or potential saving that may have accrued because of the intervention. Another study gave data for the likely savings that are possible. Elliott et al. reported an observational study that included 1524 patients with diabetes who had two or more complications and who were treated with 3-h (weekly or at longer intervals) physiologic insulin for 2 years [51]. The number of expected hospital admissions was 47 out of 100 patients per year, but only 5 were observed and the number of expected and observed emergency room visits per year was 58 and 7, respectively (*p* < 0.0001) [51].

## 6. Conclusions

Based on the evidence, loss of dynamic physiologic insulin signaling plays a major role in the pathophysiology of insulin resistance (IR). Given that IR is the accepted basis for type 2 diabetes. It, therefore, seems logical that the treatment of type 2 diabetes would be improved by switching from standard insulin treatment to a treatment that bio-mimics the normal physiologic insulin signaling process. Skjaervold et al. have been exploring the pharmacology of intravenous physiologic insulin administration as a prelude to a closed-loop intravenous insulin pump to replace the insulin pumps presently available that use a constant infusion of insulin administered by a subcutaneous route [57]. One can imagine that the next step in such a progression will be the inclusion of glucagon pulses in between the insulin pulses to further mimic the physiology of human insulin and glucagon secretion. 

We firmly believe that the evidence supports the assertion that physiologic insulin secretion is crucial in the maintenance of normal cellular insulin sensitivity. Hence, using physiologic insulin resensitization is a logical approach to restoring normal insulin function. The case studies and clinical trials examining efficacy presented in this paper are insufficient to prove the hypothesis that biomimicry of the physiologic insulin administration in this manner is broadly efficacious. Randomized clinical trial are needed. However, these reports and studies have consistently shown improvement in the usually refractory conditions that are associated with diabetes. Moreover, they demonstrate that physiologic insulin resensitization can affect several of the untoward manifestations of diabetes and, thus, appears to address the root causes of IR. They also suggest that the complications, hospitalizations, medication costs, and emergency room visits may be reduced using physiologic insulin resensitization (Table 2 and Table 3). 

This needs further research that examines the treatment’s effect on a broad array of diabetes complications. These studies should also include examining the cost of the treatment versus the costs avoided by it. If randomized controlled studies replicate the outcomes of case reports and studies examined in this review, administration of insulin in a physiologic manner represents a promising approach to reduce or avoid the looming increases in disease, disability, death and cost that will occur as the 88 million pre-diabetics progress to overt diabetes in the United States.

## Figures and Tables

**Table 1:** Summary of benefits from physiologic insulin resensitization in treating diabetic nephropathy.

Improvements in the Progression of Diabetic Nephropathy	
Halting the Progression of CKD: CrCl (18 months) [52]	348%
Reversals of CKD: Improved EGFR (3.75 months) [53]	44%
Reversals of CKD: Improved EGFR (3 months) [54]	12%

**Table 2:** Clinical Outcomes Utilizing Physiologic Insulin Resensitization.

Decreases in Hemoglobin A1c [49,58]
Reversals of Diabetic Neuropathy [49]
Improvements in Wound Healing [49]
Decreases in Insulin Requirements [49]
Improvements in Estimated Glomerular Filtration Rate (eGFR) [53,54]
Decreases in Systolic Blood Pressure (SBP) [54]
Reduce/Arrest Progression of Diabetic Nephropathy [49,52,54,58]

**Table 3:** Summary of study results discussed in this article.

Reference	Finding	Study Design	Results
Tucker et al.	Neuropathy	Case Series	Improved; discontinued Gabapentin
Tucker et al.	Foot Ulcer	Case Series	Healed quickly
Tucker et al.	HbA1c	Case Report	HbA1c decreased 2.8
Elliott et al.	Foot Ulcer	Case Series	Healed 1/3 more quickly
Dailey et al.	Nephropathy	Controlled Trial	Improved (*p* = 0.0144)
Elliott et al.	Neuropathy Pain	Case Series	93% improved, 47.5% resolved
Villaverde et al.	Nephropathy	Case Series	41% increase in GFR
Manessis et al.	Nephropathy	Case Series	12% increase in GFR
Quach et al.	Nephropathy	Case Series	11% increase in GFR
Dailey et al.	Nephropathy	Controlled Trial	Reduced decline in GFR
Aoki et al.	HbA1c	Case Series	HbA1c decreased by 1.5 T1D
Aoki et al.	HbA1c	Case Series	HbA1c decreased by 1; improved glycemic control
Elliott et al.	Hospitalizations	Case Series	Reduced hospitalizations

## Data Availability

Not applicable.
